# Distinguishing cell shoving mechanisms

**DOI:** 10.1371/journal.pone.0193975

**Published:** 2018-03-12

**Authors:** Pingyu Nan, Darragh M. Walsh, Kerry A. Landman, Barry D. Hughes

**Affiliations:** School of Mathematics and Statistics, University of Melbourne, Victoria 3010, Australia; Seoul National University College of Pharmacy, KOREA, REPUBLIC OF

## Abstract

Motivated by *in vitro* time–lapse images of ovarian cancer spheroids inducing mesothelial cell clearance, the traditional agent–based model of cell migration, based on simple volume exclusion, was extended to include the possibility that a cell seeking to move into an occupied location may push the resident cell, and any cells neighbouring it, out of the way to occupy that location. In traditional discrete models of motile cells with volume exclusion such a move would be aborted. We introduce a new shoving mechanism which allows cells to choose the direction to shove cells that expends the least amount of shoving effort (to account for the likely resistance of cells to being pushed). We call this motility rule ‘smart shoving’. We examine whether agent–based simulations of different shoving mechanisms can be distinguished on the basis of single realisations and averages over many realisations. We emphasise the difficulty in distinguishing cell mechanisms from cellular automata simulations based on snap–shots of cell distributions, site–occupancy averages and the evolution of the number of cells of each species averaged over many realisations. This difficulty suggests the need for higher resolution cell tracking.

## Introduction

Cellular migration in living tissue necessarily involves the motile cell interacting with other cells that compete with it for space and potentially impede its motion. Successful migration requires the displacement of other cells and may require remodelling of extracellular matrix. Fully detailed modelling of such processes requires attention to chemical and mechanical signals between the motile cell and its environment and the shapes of the motile cell and its neighbours. In contrast, simpler models are capable of providing insights into these subtle and complex problems. Agent–based models are especially useful, as they enable various model effects to be incorporated in a relatively simple way, facilitating *in silico* experiments related to morphogenesis and colonisation in embryonic development [1, 2], wound healing [3], and tumour growth and metastasis in cancer [4–7]. An example of the utility of agent–based *in silico* modelling to the understanding of diseases is summarised in Landman et al. [8] where the incomplete invasion of the embryonic gastrointestinal mesenchyme by neural crest cells deprives the distal intestine of neurons, a condition called Hirschsprung’s disease. A mathematical model of cell invasion, where motile cells also proliferate, successfully predicted invasion outcomes to imagined manipulations that were later verified experimentally.

It is important to emphasise that the complexity of biological processes demands that careful attention is paid to model selection before attempting to simulate biological processes computationally. It particular, the model chosen must be capable of capturing the essence of the process being studied. It is also important to know whether there is any redundancy. Knowing which features of the model may be discarded and still yield satisfactory concordance with experimental observations gives important information not only on the model chosen, but also on the biological process and the sensitivity of the experimental measurements to capture the process of interest. In this study we will examine the ease with which different agent–based motility mechanisms can be distinguished using metrics closely related to biological measurements.

A motivating example for our approach is the experimental work reported by Iwanicki et al. [9] and Davidowitz et al. [10]. They studied an *in vitro* invasion process in which small clusters of ovarian cancer cells placed on top of an epithelial cell monolayer (grown on a suitable tissue culture substrate) force their way into the epithelial cell layer. This is a simple example of a more general problem in which a relatively thin layer of tissue is invaded by motile cells. We do not purport to model the ovarian cancer cell experiments specifically here, but rather to investigate more broadly model selection and redundancy for invasion problems. If we were concerned with detailed modelling of invasion into tightly constrained tissue, for which cells undergo large deformation and squeeze through interstices rather than moving into vacant space or simply displacing other agents, or use of structureless agents to represent cells would be an excessively crude approximation.

Although invasion processes can be modelled using deterministic equations in which space and time are continuous, such approaches cannot shed light on the extent of variability in outcomes in the presence of the very real spatial and temporal stochasticity of motile biological cell populations. In contrast, each *in silico* experiment on an agent-based model shows the locations of all cells in the model system. Averaging over large numbers of *in silico* experiments with agent-based models gives access to similar information to that which one can obtain by deterministic continuum modelling (see the Appendix).

There have been many recent papers on agent–based models with potential application to development or invasion processes implemented on regular lattices. Typically, such models involve randomly moving agents (representing cells) subject to an exclusion process [11] in which attempted agent moves that would place an agent on an already occupied site are aborted. The probabilities of selection of which moves are to be attempted can also be allowed to depend in some way on the occupancy status of other sites in the vicinity of a site that is selected at random to attempt to move. This framework has also been extended to allow for multiple cell species [12].

In exclusion processes of the type just described, invasion into regions of high agent density is essentially ruled out by the abortion of all but very few attempted moves, and such models give little insight into the class of problems that we are studying here. However, in the context of real tissue invasion processes, invading cells can force other cells out of the way, so we turn to a class of agent–based models in which an agent attempting to move in a given direction may be permitted to shove other agents out of the way to create a vacant site that it can proceed to occupy. The prototypical model of this class was studied by Almet et al. [13]; see also related work in the context of biofilms [14–16] and the paper by Yates et al. [17]. Almet et al. [13] analysed the (simple) shoving mechanism in three different scenarios: single species shoving, multi-species shoving and a mixed scenario where resident cells could not shove other cells (these cells were called “gentlemen”) but any invading cell could shove other cells (these invading cells were called “ruffians”). Using a mean-field approximation for site occupancy, the continuum limit yielded a nonlinear diffusion equation in each scenario which was compared to average site occupancy from many realisations of the agent–based model.

Almet et al. [13] found that the shoving mechanism allows agents to spread faster than the simple exclusion motility rule as fewer moves are aborted. The agreement between average site occupancy over many realisations and the continuum limit PDEs (nonlinear diffusion equations for single species and advection-diffusion equations for multiple species systems) was poor in lattices of one spatial dimension but very good in two dimensions, since on a two-dimensional lattice cells may move around each other by taking vertical moves. The absence of this possibility in one dimension renders the mean-field approximation invalid.

Yates et al. [17] also implemented a single species shoving mechanism, where each cell can shove *K* neighbouring cells in order to move one space into a chosen occupied site. They found good agreement between their agent–based model and the numerical solution of the continuum limit mean–field PDE for all *K* ∈ [0, 4], though there was a slight increase in the error as *K* increased.

It was noted in Almet et al. [13] that the implemented shoving mechanism might usefully represent the mechanism responsible for ovarian cancer cells shoving mesothelial cells out of the way in Iwanicki et al. [9]. We will be particularly interested in the ruffians and gentlemen scenario as a model of invading cells (shovers) colonising a high cell-density tissue (composed of gentleman cells). We shall always colour the invading cells as red (this has the mnemonic value that the invading cells would be considered as dangerous in the context of cancer) and the cells originally present in green.

Our goal is to determine how much information on the nature of the shoving mechanism can be gleaned from agent–based simulations of shoving mechanisms. We attempt to distinguish between shoving mechanisms based on simulation results that mimic typical biological observations, such as the time–lapse images of mesothelial cell clearance in Iwanicki et al. [9] or the evolution of the number of cells of each species under investigation. We have discovered that it is difficult to distinguish between the simple and smart shoving motility mechanisms by merely examining single realisations of an invasion or from the evolution of averaged cell counts of each species. We therefore emphasise the need for caution in choosing a particular shoving mechanism to model cell behaviour, since differences between mechanisms may not be revealed by particular experimental measurements.

## 1 The agent–based model

We consider a finite square lattice, with dimensions *L*_*x*_ × *L*_*y*_ = 200 × 20, for which sites have integer valued coordinates (*x*, *y*), where 1 ≤ *x* ≤ *L*_*x*_ and 1 ≤ *y* ≤ *L*_*y*_. Each site may be occupied by at most one agent and we now describe how the occupancy of sites evolves as the integer-valued time coordinate increases. Those agents that are present at time *t* = 0 will be called *green agents* and will be denoted by the letter *G*. Their uniform initial concentration is denoted by *c*_*G*_, where 0 ≤ *c*_*G*_ ≤ 1. Any agents that are introduced at subsequent times are called *red agents* and will be denoted by the letter *R*. Such agents are interpreted as invaders, and may displace green agents. (Where necessary, *R* and *G* will also refer to the concentrations of each cell species).

**Fig 1:**
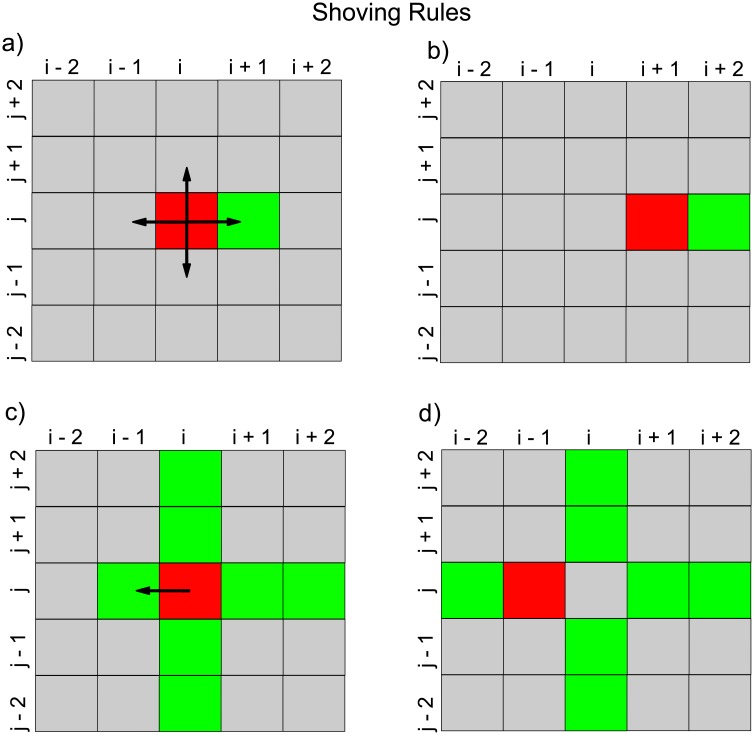
A schematic diagram which distinguishes between simple and smart shoving agents. Occupied locations are coloured either red or green. Agents which may shove obstructing agents are displayed in red, while the occupied sites which obstruct the possible movements of the shoving cells are displayed in green. a) A simple shoving agent has a choice of four locations to move to, indicated by arrows. It chooses one of these directions at random. b) If the simple shoving agent at location (*i*, *j*) chooses to move to location (*i* + 1, *j*), it moves to this location, pushing the obstructing cell at (*i* + 1, *j*) to location (*i* + 2, *j*). c) A smart shoving agent is located at (*i*, *j*). d) The smart shoving agent at (*i*, *j*) chooses to move to the location that expends the least amount of shoving effort. It will move from (*i*, *j*) to (*i* − 1, *j*) and will push the obstructing agent at (*i* − 1, *j*) to location (*i* − 2, *j*). Moving in any other direction would expend more shoving effort than the chosen direction (i.e. it would require pushing two obstructing cells rather than one).

*Motility mechanism*. Within the lattice, at each time *t*, if there are currently *N* agents (ignoring colour) within the system, we make *N* sequential independent choices of agent, so that on average each agent is chosen once per time step, although an individual agent may be selected several times or not selected at all on a given time step. A selected agent is given the opportunity to attempt a move of one lattice space immediately on being selected. The way in which it attempts a move depends on its personality: all agents of the same colour possess the same personality, but there are three personality types: *gentlemen*, and two types of ruffian, viz. *simple shovers* and *smart shovers*. The motility behaviours that distinguish between simple and smart shoving agents are summarised in Fig 1.

1. *Gentlemen agents* If a gentleman is selected to move, it selects one of the four directions up, down, left or right at random as its attempted direction to move to a target site. If a move of one lattice spacing in the selected direction would place the cell on a target site that is not currently occupied, then the move is made, but if the target site is already occupied, the movement attempt is aborted.
2. *Simple shoving agents*. If a simple shover is selected to move, it selects one of the four directions up, down, left or right at random as its attempted direction to move to a target site. If the target site is vacant, it moves to occupy it. If the target site is occupied, the simple shover attempts to push the occupant out of the way. The attempt is always successful if the move to the target site is a horizontal move. For example, when the simple shover moves to the right, it forces the present occupant of the target site and all other agents that lie between the present occupant and the first vacant site on the right of the simple shover to move to the right also. This may lead to an agent exiting the lattice at the right vertical boundary. However, if the attempted move is vertical (up, say), shoving the present occupant (and any other agents that need to be displaced) out of the way only succeeds when there is at least one vacant site between the simple shover and the upper horizontal boundary. If there is no vacant space, then the attempt to move is aborted.
3. *Smart shoving agents*. If a smart shover is selected to move, it looks at the four directions and determines which move will displace the least number of cells. If there are several such moves to choose from, one is chosen at random. If a vertical move is not permitted, due to there being no available sites between the chosen site and the horizontal boundary, the smart shover will choose a horizontal move instead. Therefore smart shovers never abort moves, unlike simple shovers (whose vertical move attempts fail if there are no vacant locations between them and the boundary) or gentlemen cells (whose attempts to move onto any occupied site always fail).

*Injection mechanism*. We will be concerned with different cell types invading a domain, which will be vacant in Section 2.1 and occupied by a uniform distribution of gentlemen (green) cells in Section 2.2. The injection protocol mirrors the shoving capability of the invading cell. Four cells attempt to enter the domain per time–step. Each invading cell chooses a location in the invasion region and attempts to occupy it. If it is empty, it will occupy this site. If the chosen site is occupied, the character of the invading cell becomes important. If the invading cell is a gentlemen cell, the invasion attempt is aborted. If the invading cell is a simple shover, the cell randomly chooses a direction to shove the resident of the occupied cell (and any other cells neighbouring it). If the chosen direction is horizontal, the attempt is always successful. If it is vertical, the attempt is aborted if there are no vacant sites between the chosen occupied site and the horizontal boundary in the direction of the shove. As noted above, the permissibility of horizontal shoves ensures that invasion attempts by smart shoving cells are always successful.

## 2 Model distinguishability

The experiments of Iwanicki et al. [9] suggest that ovarian cancer spheroids invade the submesothelial layer by exerting a force on the mesothelial cells lining an organ and clearing them from the locality. They called this mechanism mesothelial cell clearance (a schematic of this mechanism may be found in Iwanicki et al. [9]). No previously presented simple agent–based models for motile cell systems other than those of Almet et al. [13] and Yates et al. [17] have the ability to clear a cell from its current location and shove it to a neighbouring location. A key concern of our modelling is to determine the extent to which the way shoving is implemented can be discerned from simple observations of the model output. We focus on the distinction between purely random shoving (the simple shover model) and more strategic shoving (the smart shover model).

The smart shover rule allows a cell that is crowded to respond to the wider environment rather than solely to those cells in a randomly chosen direction immediately adjacent to it. The rule can be interpreted in various ways. For example, it can be taken as a de facto representation of the unequal resistance encountered by the invader on pushing against adjacent cells in different directions. It is useful to consider smart shoving as an alternative to the extreme cases of strict exclusion and simple shoving, in which all attempts to move are made without use of any knowledge of the local environment (and in some cases are therefore aborted, when an alternative move might have been possible).

**Fig 2:**
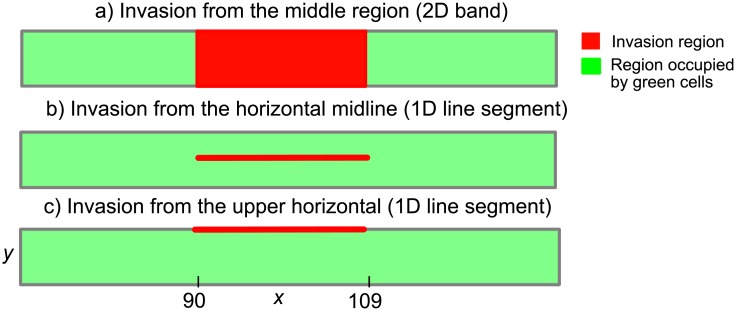
Schematic of the three invasion regions considered. Invasion regions are displayed in red, whereas a background of resident cells is displayed in green. When considering invasion of red cells into an empty domain, the uniform concentration of the green cells is *c*_*G*_ = 0. When considering invasion of red cells into a non-empty domain, the uniform concentration of the green cells is taken to be *c*_*G*_ = 0.6, unless otherwise stated. The domain has dimensions *L*_*x*_ × *L*_*y*_ = 200 × 20. In a) the red cells enter the lattice from a 2D band in the middle region of the lattice defined by coordinates *x* ∈ [90, 109] and *y* ∈ [1, 20]. In b) the red cells enter the lattice from the horizontal midline of the lattice defined by coordinates *x* ∈ [90, 109] and *y* = 10. In c) the red cells enter the lattice from upper horizontal boundary of the lattice defined by coordinates *x* ∈ [90, 109] and *y* = 20. Homogenous vertical and reflecting horizontal boundary conditions are implemented in all simulations of the agent–based model. In all simulations, invasion of red cells occurs at a rate of 4 attempts per time-step.

Time-lapse imaging of a single experiment corresponds to a following the time evolution of a single realisation of an agent–based model. We shall investigate whether we can distinguish between our shoving models (and indeed invaders of the gentlemen type) from snapshots of invading and resident cell distributions at specific times. Although it is frequently not practicable to repeat identically prepared biological experiments hundreds of times, we shall also investigate the extent to which averaging cell counts from many realisations of our models enables us to distinguish clearly between these models. To guard against drawing conclusions from a single geometrical context, we consider three different locations of the invasion region (the source of the invading agents). A schematic displaying these choices for the invasion region is shown in Fig 2. The green areas represent the background of resident cells, if present, which the invading cells diffuse into from the red invasion region.

### 2.1 Red cells invade an empty domain

Before considering the forced displacement of incumbent cells from a layer by invading cells, we consider the case where the invaders enter virgin territory, to benchmark those dynamical effects that are associated purely with the invaders themselves. We seek to distinguish between our simulations of the three motility mechanisms using only the three simulation observables (snap–shot of cell distribution at fixed times, average site-occupancy over 300 independent realisations and the evolution of the cell count of each species averaged over 300 realisations). Each observable is considered separately below.

#### 2.1.1 Observable 1: Snap–shots of cell distribution at times *t* = 50 and *t* = 250

**Fig 3:**
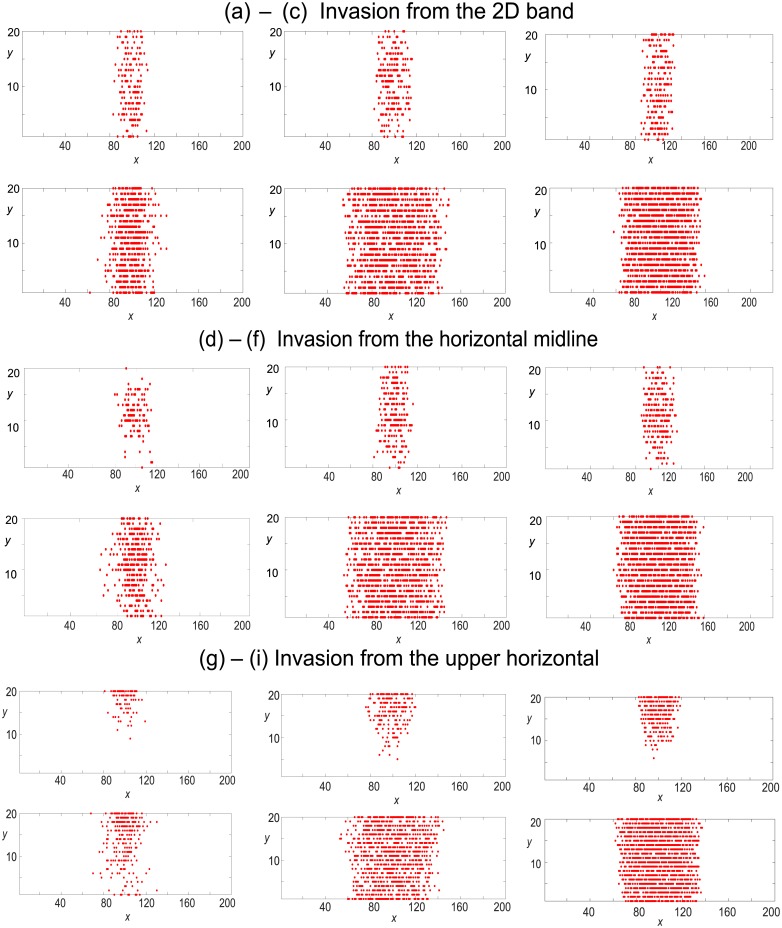
Single realisations of the agent–based model displaying snap–shots of cell distributions from invasion of an empty domain from three separate locations (displayed in Fig 2). Red cells enter the domain at a rate of four attempts per time-step. Simulations are displayed at times *t* = 50, 250. The first column denotes gentlemen invaders, the middle column denotes simple shover invaders and the third column denotes smart shover invaders.

In Fig 3 we analyse whether the three motility mechanisms (gentlemen, simple and smart shovers) can be distinguished from single realisations of our agent–based model when the cells invade an empty domain. Varying the location of the invasion region allows us to examine the interaction of each mechanism with the geometry of the domain. Since gentlemen invaders are most susceptible to volume exclusion, which may cause invasion attempts to be aborted, we find that the number of gentlemen cells is highest when the area of the region of invasion is largest (invasion from a 2D band). Crowding of the invasion region reduces gentlemen cell number when the invaders enter from the horizontal midline, Fig 3(d), and this effect is exacerbated by the reflecting horizontal boundary condition when invaders enter from the upper horizontal boundary, Fig 3(g). The cell distribution patterns resulting from simple and smart shoving invaders (columns two and three) are broadly similar. They display a larger cell count and cells travel further from the invasion regions than gentlemen invaders, though the distribution of smart invaders is more compact than that of the simple shovers since smart shoving cells shove in the direction that expends least shoving effort.

#### 2.1.2 Observable 2: Site-occupancy averages

**Fig 4:**
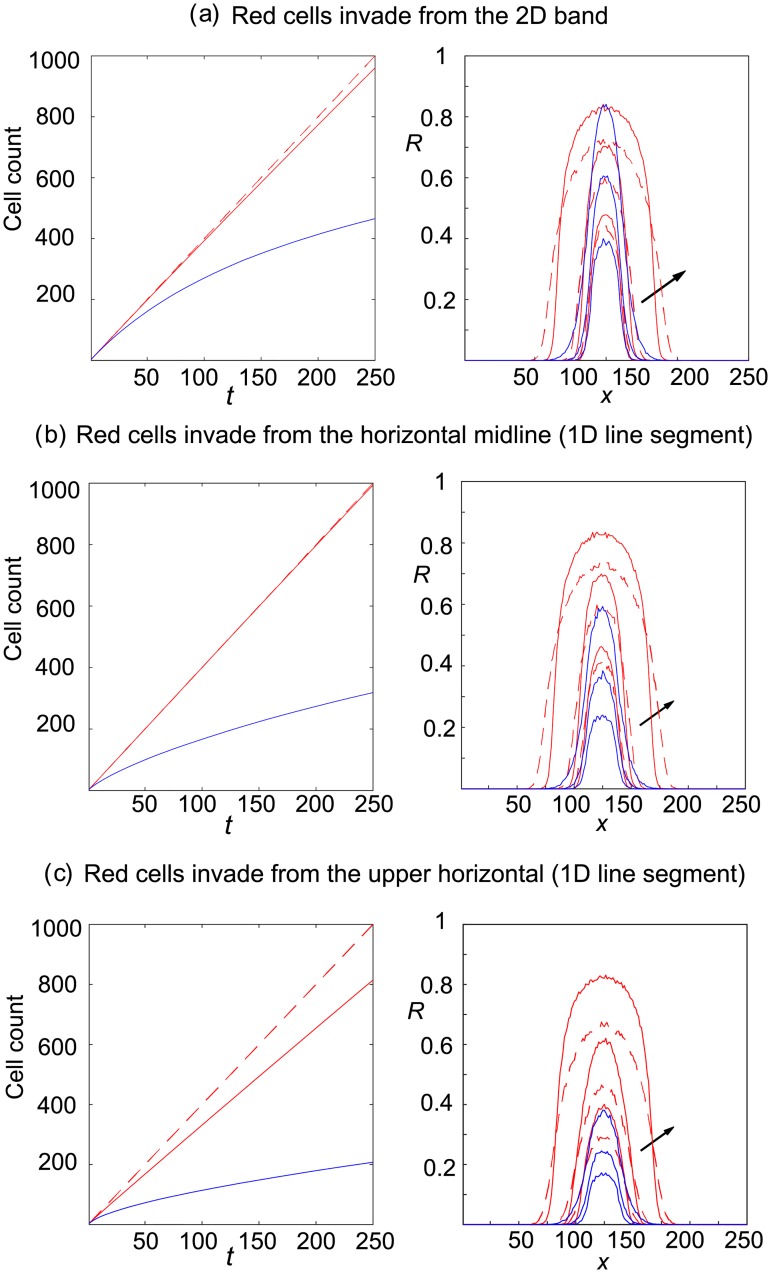
Site occupancy averages from invasion of an empty domain from three separate locations (displayed in Fig 2). Red cells enter the domain at a rate of four attempts per time-step. Site occupancies obtained from the agent–based model are averaged over 300 independent realisations at times *t* = 50, 100, 250. The continuous red curve denotes simple shovers, a broken red curve denotes smart shovers and a continuous blue curve denotes invading gentlemen cells. Arrows denote the direction of cell movement as time increases.

We now move from observables based on single realisations of the agent–based model, to observables obtained by averaging over 300 realisations. It is clear from the second column in Fig 4 that invading smart shovers create a more compact cell distribution than simple shovers, which spread further from the invasion region in each invasion region scenario. The role of the invasion region in exacerbating volume exclusion effects for gentlemen invaders is clear from Fig 4, where increased cell crowding when invasion is from a 1D line segment (either midline or upper horizontal invasion regions) causes more invasion attempts to be aborted than when invasion is from the 2D band in the centre of the domain. Cell crowding in the invasion region is greatest in the upper horizontal invasion region scenario (due to the reflecting horizontal boundary condition) and consequently the number of gentlemen cells in this scenario is the lowest of the three invasion region scenarios. This may also be seen from the cell count curve for gentlemen invaders in Fig 4(c) below.

#### 2.1.3 Observable 3: Cell–count averages

In Fig 4 the evolution of the cell count of each species, averaged over 300 independent realisations of the agent–based model, is also tracked. As expected, the location and size of the injection region dramatically affects the number of gentlemen cells that are successfully injected. The number of gentlemen cells is highest when injection occurs from the 2D band in the middle of the domain. Crowding effects are significantly greater when injection occurs from a 1D line segment (the horizontal midline or upper horizontal injection location scenarios) and the reflecting boundary condition on the upper horizontal further increases cell crowding in this region, increasing the probability that a site selected for invasion by a gentlemen cell will be occupied and hence the injection aborted.

More simple shoving cells successfully invade from the horizontal midline than when invasion occurs from the 2D band in the middle of the lattice. This is because simple shoving cells may abort moves (and invasion attempts) if the direction they choose to push a cell is vertical and there are no empty sites between the chosen occupied site and the horizontal boundary in that direction. Aborted invasion attempts are more likely when invading simple shovers may select a site close to a horizontal boundary, where fewer sites need to be occupied to abort a move than if cells invade from the midline.

For each invasion region scenario, smart shoving invaders are most successful, given their ability to choose the direction in which to shove cells. Invasion attempts are always successful since if the vertical column of sites of a chosen invasion location is entirely full, the smart shoving cell will choose a horizontal push instead, which are always permitted.

Although we can distinguish between gentlemen invaders and shoving invaders on all three metrics (snap–shots, site-occupancy averages and average cell counts) from each invasion location, shoving mechanisms are indistinguishable on the basis of snap–shots of cell distributions. We may distinguish between shoving mechanisms based on site–occupancy averages (since smart shovers yield more compact patterns from all invasion locations) and on the basis of averaged cell counts if invasion is restricted to occurring from the upper horizontal boundary. many independent realisations of the agent–based model.

### 2.2 Invading an already occupied domain

From a biological perspective, we are most interested in the invasion of a dense collection of cells (‘tissue’) by cancer cells which have the capacity to shove incumbent cells out of their way to reach their desired location. This is the scenario that most closely models the *in vitro* experiments in Iwanicki et al. [9], as suggested by Almet et al. [13].

The results of the previous section tell us that it is difficult to distinguish between shoving mechanisms by examining single snap–shots of cell distributions or on the basis of the average number of cells of each species (unless invasion is from the upper horizontal boundary). We now consider whether cell migration mechanism distinguishability is aided or hindered when we add a background of green resident gentlemen cells with uniform density *c*_*G*_ = 0.6. Again we examine simulation results for each observable in turn and draw conclusions at the end of the section.

#### 2.2.1 Observable 1: Snap–shot of cell distribution at times *t* = 50 and *t* = 250

**Fig 5:**
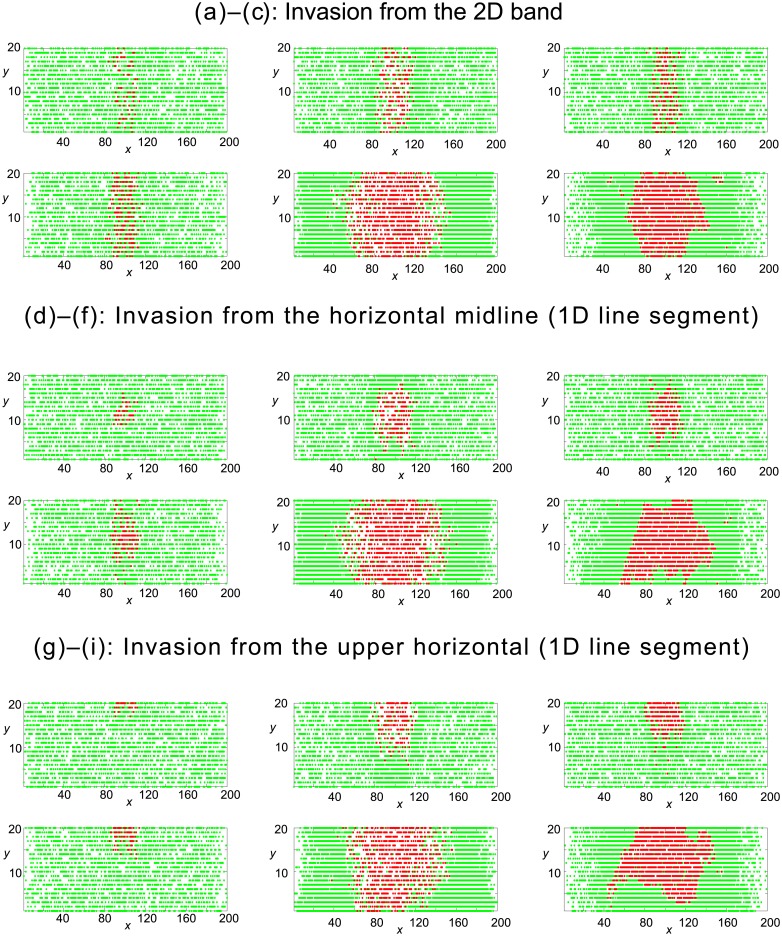
Single realisations of the agent–based model describing invasion of an occupied domain from three separate locations (displayed in Fig 2). Green resident cells are gentlemen in each scenario (with initial uniform concentration *c*_*G*_ = 0.6). Red cells enter the domain at a rate of four attempts per time-step. Simulations are displayed at times *t* = 50, 250. The first column represents gentlemen invaders, the middle column represents simple shover invaders and the third column represents smart shover invaders.

In Fig 5 we examine whether cell mechanisms can be distinguished via snap–shots of cell distributions at times *t* = 50 and *t* = 250. All background green cells are gentlemen cells. Similar to our results on invasion of an empty domain in Fig 3, we see that gentlemen cells are less likely to invade the domain successfully, owing to volume exclusion and their inability to shove neighbouring cells. Simple shoving invaders successfully invade and push green cells out of the invasion region. The distribution pattern created by smart shoving cells is more compact with few empty or green sites remaining in the invasion region.

#### 2.2.2 Observable 2: Site–occupancy averages

**Fig 6:**
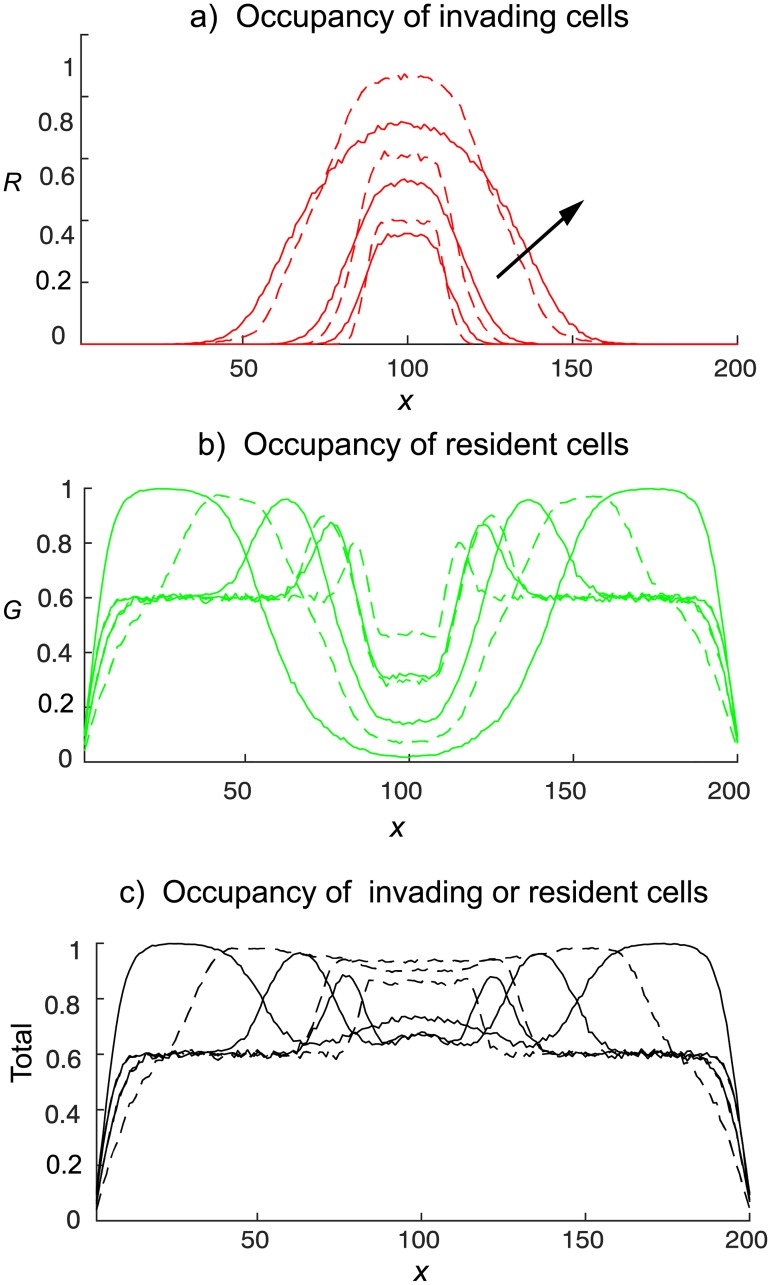
Invasion of an occupied domain from the 2D band (displayed in Fig 2(a)). Green gentlemen cells are present with density *c*_*G*_ = 0.6. Red cells enter the domain at a rate of four attempts per time-step. Site occupancies, obtained from the agent–based model, are averaged over 300 independent realisations at times *t* = 50, 100, 250. The continuous curves denotes simple shovers, and broken curves denotes smart shovers. Arrows show increasing time.

We now compare the cell distributions resulting from the interaction of invading cells with a non-shoving background of green resident gentlemen cells for each red cell motility mechanism. Simulation results, averaged over 300 independent realisations of the agent–based model, for shovers invading gentlemen resident cells from the 2D band in the centre of the domain are given in Fig 6.

**Fig 7:**
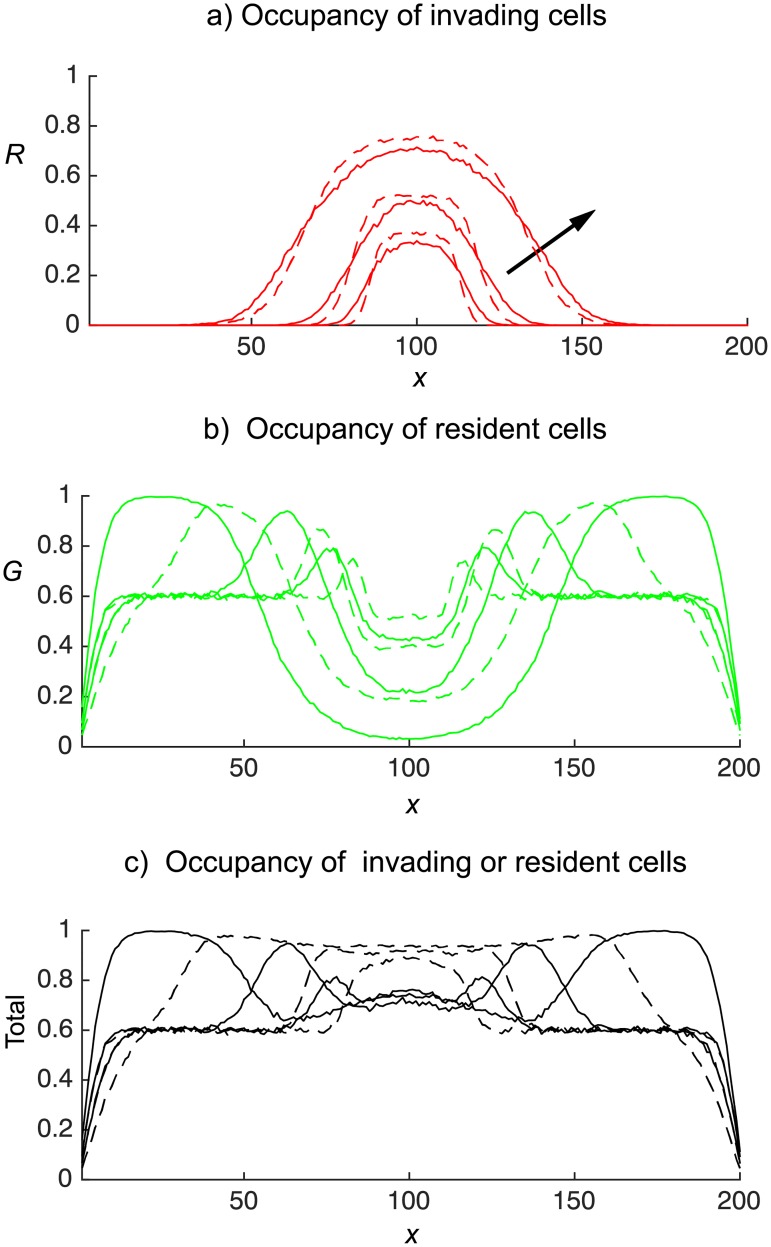
Invasion of an occupied domain from the horizontal midline (displayed in Fig 2(b)). Green gentlemen cells are present with density *c*_*G*_ = 0.6. Red cells enter the domain at a rate of four attempts per time-step. The continuous curves denotes simple shovers, and broken curves denotes smart shovers. Site occupancies, obtained from the agent–based model, are averaged over 300 independent realisations at times *t* = 50, 100, 250. Arrows show increasing time.

**Fig 8:**
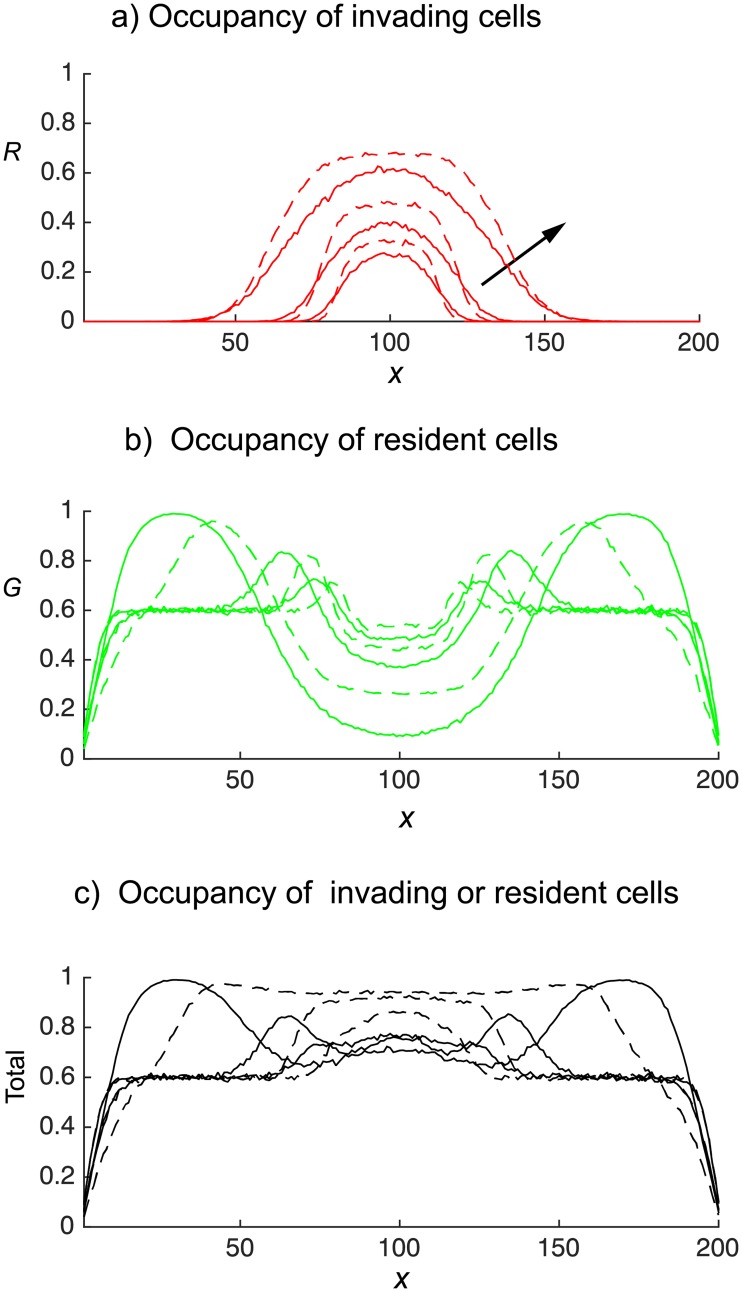
Invasion of an occupied domain from the upper horizontal (displayed in Fig 2(c)). Green gentlemen cells are present with density *c*_*G*_ = 0.6. Red cells enter the domain at a rate of four attempts per time-step. The continuous curves denotes simple shovers, and broken curves denotes smart shovers. Site occupancies, obtained from the agent–based model, are averaged over 300 independent realisations at times *t* = 50, 100, 250. Arrows show increasing time.

From Fig 6 we clearly see that the gentlemen green cells, initially having uniform concentration of 0.6, are being shoved out of the middle invasion region towards the left and right boundaries by the invading shover cells, which come to dominate to middle region (Fig 6(a)). The simple shover invading cells (continuous red curve) spread further from the invasion region than the smart shover invading cells (broken red curve). Furthermore, the unlimited potential of simple shovers to push cells horizontally causes the occupancy of the area close to the vertical boundaries to be much higher than the corresponding areas resulting from smart shoving invaders. This leads to a central region that is significantly more dense when invaders are smart shovers as compared to when invaders are simple shovers, see Fig 6(c). Similar conclusions may be drawn when invasion is from the horizontal midline, displayed in Fig 7, and when invasion is from the upper horizontal boundary, displayed in Fig 8.

In Fig 6, we see that the single realisation patterns derived from our agent–based model, displayed in Fig 5(b) and 5(c), also generate a consistent pattern when averaged over many realisations.

#### 2.2.3 Observable 3: Cell–count averages

**Fig 9:**
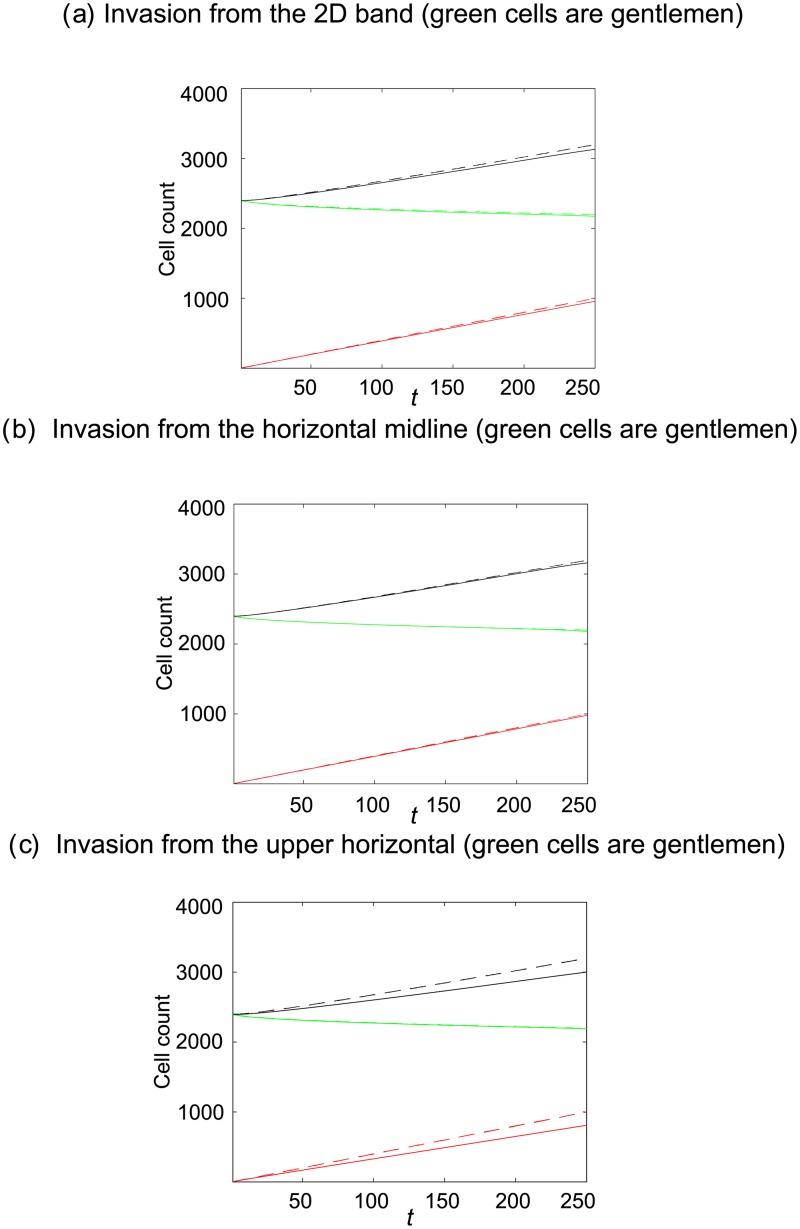
Cell counts, averaged over 300 realisations of the agent–based model, from invasion of an occupied domain (uniformly seeded green gentlemen cells with cell density *c*_*G*_ = 0.6). Continuous lines denote the scenario where invaders are simple shovers, whereas broken lines denote the scenario where invaders are smart shovers. Red cells enter the domain at a rate of four attempts per time-step. The red lines denotes the number of red cells present, the green lines denote the number of green gentlemen cells present and the black lines denote the total number of cells present.

In Fig 9 we see that cell counts resulting from invading simple and smart shovers are practically indistinguishable in the 2D band and horizontal midline invasion region scenarios, Fig 9(a) and 9(b), though crowding effects leads to differences in cell count averages when invasion is from the upper horizontal, Fig 9(c). These results mirror those found with invasion of an empty domain in Fig 4. Although we can distinguish between the averaged site occupancy patterns resulting from simple and smart invading cells, see Figs 6 and 8, the loss of spatial information has prevented us from distinguishing between the two shoving mechanisms on the basis of averaged cell counts of each species, except when invasion is from the upper horizontal. This result emphasises that cell motility mechanisms distinguishable on the basis of one metric may not be distinguishable on the basis of another metric.

In summary, the conclusions drawn from vacant invasion scenarios, namely that only gentlemen invaders may be easily distinguished on the basis of snap–shots of cell distributions, averaged site-occupancies and averaged counts of each cell species, are still valid. The presence of a background of cells does not help to distinguish between shoving cell mechanisms, except in the case of distinguishing on the basis of averaged site-occupancy, where a compact region in the centre of the domain results from smart shoving invaders.

## 3 Discussion

We have analysed a new mechanism for cell motility, which we call ‘smart shoving’, whereby a cell makes motility choices that minimise the shoving effort expended. We have examined the multi-species interaction of invading cells with cells initially present, where each cell species can either shove neighbouring cells out of the way (via simple or smart shoving rules) or where a choice to move to an occupied site leads to abortion of the move. Considerable redundancy was found when comparing simulation results from each cell-type interaction on typical metrics (such as snap–shots of single realisations of the cell distribution, the evolution of the average counts of each cell species and the averaged distribution of site occupancies). It was difficult to distinguish between either cell shoving mechanism on the basis of single realisations or averaged evolution of cell counts (except in the most restrictive case where invasion is from the upper horizontal boundary), which points to a need for a combination of measurements to distinguish cell motility mechanisms.

Numerous studies have reported the insensitivity of agent–based algorithms to biologically important aspects of the cell behaviour being modelled. For example, Cai et al. [18] sought to distinguish between inhibitory and repulsive effects, produced by the signalling protein Slit, on the migration of neurons. Various cell-sensing models were compared by examining the individual cell trajectories they produced and the collective behaviour that resulted from population averaging. Each model was then compared to experimental data in the hope that this inverse modelling approach could infer the role of Slit on cell migration, inhibitory or repulsive. Their approach, like ours, sheds light on whether the rules of migration (for example transition probabilities) can be deduced from time–lapse images measuring individual cell path-lines and cell distributions.

Cai et al. [18] found that whilst certain general features of the neuronal response to Slit could be deduced (e.g. if there is a gradient in the signalling molecule, an asymmetric cell distribution should result), the specific cell-sensing response could not be deduced without considering cell count numbers, cell displacements over small enough time intervals and cell position before and after introduction of Slit. This conclusion suggested future experimental refinements needed to isolate the specific migration mechanisms at play from the inevitable noise inherent in complex biological systems.

In cancer cell modelling, cell proliferation is particularly crucial. Whilst our simulations did not include cell proliferation, there is evidence that suggests its inclusion would further complicate the task of distinguishing cell motility mechanisms from time–lapse imaging, such as in Iwanicki et al. [9]. Simpson et al. [19] studied the invasion of motile cells that could also proliferate and found that the the invasion wave spread was relatively insensitive to the particular motility mechanism. Thus, little useful information regarding a cell’s motility mechanism could be gleaned from the standard experimental measurement of a cell population’s invasion wave speed. Furthermore, it was suggested by Simpson et al. [20] that wavefront width data and time–lapse images which capture proliferation events should also be measured before proliferation mechanisms can be adequately distinguished.

By emphasising the insensitivity of simulation studies to particular motility mechanisms, these simulation studies have proven invaluable in clarifying the experimental measurements which would be most most useful to distinguish cell motility mechanisms. A smart-shoving model is more plausible than a simple shoving model as a first approximation to describing an invasion mechanism in which invading cells have to push through an existing population, but it is difficult to distinguish between the processes on the basis of single realisations of each. High-resolution cell tracking in the laboratory seems an essential prerequisite for useful model selection in invasion processes.

A clear conclusion from our modelling is that caution must be exercised in the choice of motility/shoving mechanism to endow cells with. This study reveals the need for time–lapse imaging capturing cells at shorter time intervals than typically presented. An intriguing possibility would be to utilise the transparency of zebrafish, for example, to record migrating cells. Live imaging of this species, whose embryos are transparent, has yielded many fundamental insights which are hidden in static time–lapse imaging, notably the dynamic role of oligodendrocyte cells in myelination Czopka et al. [21]. A salient extreme example of the advantages of live imaging may be found when considering the challenges encountered by Walsh et al. [22], who developed an agent–based computational model to test whether observed patterns of oligodendrocytes in the mouse corpus callosum were due to cell migration or proliferation. The need to sacrifice the mice to measure cell counts and patterns at each time point introduced variation into the measurements, which made the task of deciphering mechanisms more challenging.

Minimising the interval between time–lapse images may capture the key diagnostic features of a motility mechanism, which we have shown are difficult to distinguish in focused simulations owing to mechanism redundancy. This would lead to more informed modelling with minimal mechanism uncertainty and allow modelling approaches to consider more complex biological systems.

## Appendix: Comparing simulations with solutions of a mean-field-based PDE

We derive the continuum limit PDE system (in the mean-field approximation) of the invasion scenario with gentlemen resident cells and simple shover invading cells and then compare this to the averaged simulation results from our agent–based model. The continuum limit is an effective means of studying collective cell behaviour and the evolution of gradients in bulk variables such as cell concentration. The continuum equations that follow utilise the mean–field approximation, where the positions of cells are assumed to be independent. More realistic equations that incorporate correlations between cells (such as long-range interactions) are expected to reduce to the equations derived using the mean–field approximation in the low–density, weak correlation limit.

We denote the concentration of the red invading cells by *R*, the concentration of the green cells initially present by *G* and the total concentration of cells of both types by *C*. Note that *R* = 1 or *G* = 1 correspond to maximal occupancy by red or green cells, respectively.

When all agents are simple shovers, from the analysis of Almet et al. [13] (without any cells entering the system) the PDE system with *C* = *R* + *G* is given by

$$\begin{array}{c} \begin{array}{cc} \frac{\partial R}{\partial t} & =D_{0}\frac{\partial }{\partial x}(D\left(C\right)\frac{\partial R}{\partial x}+RV\left(C\right)\frac{\partial C}{\partial x})\\ \frac{\partial G}{\partial t} & =D_{0}\frac{\partial }{\partial x}(D\left(C\right)\frac{\partial G}{\partial x}+GV\left(C\right)\frac{\partial C}{\partial x}), \end{array} \end{array}$$ (1)

where

$$D\left(C\right)=\frac{1}{1-C},\;V\left(C\right)=\frac{3-C}{{\left(1-C\right)}^{3}}.$$ (2)

To relate this PDE system to our agent–based simulation model we need to add an influx of red cells. During each time step, four red cells enter the system. Therefore, we need to add a source term *S*_*R*_ (which is zero outside the invasion interval, denoted [*Z*_*l*_, *Z*_*r*_], where *Z*_*l*_, *Z*_*r*_ are positive integres) to the equation governing *R*, i.e., Eq (1) becomes

$$\begin{array}{c} \begin{array}{cc} \frac{\partial R}{\partial t} & =D_{0}\frac{\partial }{\partial x}(D\left(C\right)\frac{\partial R}{\partial x}+RV\left(C\right)\frac{\partial C}{\partial x})+S_{R}\\ \frac{\partial G}{\partial t} & =D_{0}\frac{\partial }{\partial x}(D\left(C\right)\frac{\partial G}{\partial x}+GV\left(C\right)\frac{\partial C}{\partial x}). \end{array} \end{array}$$ (3)

Four cells attempt to enter the invasion region per time-step, therefore the probability of a cell entering the invasion region is *P*_entry_ = 0.01 and the source term becomes

$$S_{R}=\frac{1}{L_{y}}P_{\text{entry}}\cdot H\left(C\right)\cdot E\left(x\right),$$ (4)

where *H*(*C*) is a step function defined as

$$H\left(C\right)=\{\begin{array}{cc} 1\; & \text{if}\;0<C<1;\\ 0 & \text{if}\;C=1 \end{array}$$ (5)

and the function *E*(*x*) is only non–zero at locations where the red cells can enter the system:

$$E\left(x\right)=\{\begin{array}{cc} 1\; & \text{if}\;Z_{l}\leq x\leq Z_{r};\\ 0 & \text{if}\;x<Z_{l}\;\text{or}\;x>Z_{r}. \end{array}$$ (6)

Note that for gentlemen invaders a factor of (1 − *C*) must be included in the definition of *E*(*x*), since invasion attempts are aborted if the chosen site is occupied.

Similarly, in the case that *G* cells are gentlemen and the *R* cells are simple shovers, the PDE system for the concentrations of each species, denoted *R* and *G* respectively, becomes

$$\begin{array}{c} \begin{array}{cc} \frac{\partial R}{\partial t}= & D_{0}\frac{\partial }{\partial x}(D\left(C\right)\frac{\partial R}{\partial x}+RV\left(C\right)\frac{\partial C}{\partial x})+S_{R}\\ \frac{\partial G}{\partial t}= & D_{0}\frac{\partial }{\partial x}\{(1-C+\frac{R}{1-C})\frac{\partial G}{\partial x}\\ & +G[\frac{3-C}{{\left(1-C\right)}^{2}}\frac{\partial R}{\partial x}+(1+V(R,C))\frac{\partial C}{\partial x}]\}, \end{array} \end{array}$$ (7)

where

$$D\left(R,C\right)=1+2R\frac{2-C}{{\left(1-C\right)}^{2}},\;V\left(R,C\right)=R\frac{3-C}{{\left(1-C\right)}^{3}},$$ (8)

where the function *S*_*R*_ is given by (4). Setting *S*_*R*_ = 0 we recover the equations described in [13].

The PDE equations are solved numerically using MATLAB pdepe, which calls the stiff solver ode15s with adaptive time stepping. The boundary conditions imposed on the vertical boundaries are homogenous, with the left boundary *x* = 0 and right boundary *x* = *L*_*x*_, respectively. The grid spacing is *δx* = 0.1 and the results were checked to be grid-independent.

In other agent–based systems with volume exclusion effects, the continuum limit PDEs obtained from mean-field arguments have often been found to give good predictions of the results from averages over a large number of realisations, especially at low densities. Though the initial concentration of both compact ruffian (simple shover) and gentlemen cell species was 0.75 in Fig 6 of [13], the otherwise empty domain facilitated the spread of cells of both species resulting in a diffusive spread of each species from initial high concentration to lower concentrations. We have found that when the green cells are initially distributed uniformly, the invading red cells shove them into higher concentrations to the left and right of the injection region. In Fig 6(b) we see that green cells approach full occupancy to the left and right of the injection region by *t* = 50 when the initial concentration of green cells is initially *c*_*G*_ = 0.6. The appearance of such high density regions signals the unsuitability of any mean-field based PDE approach.

**Fig 10:**
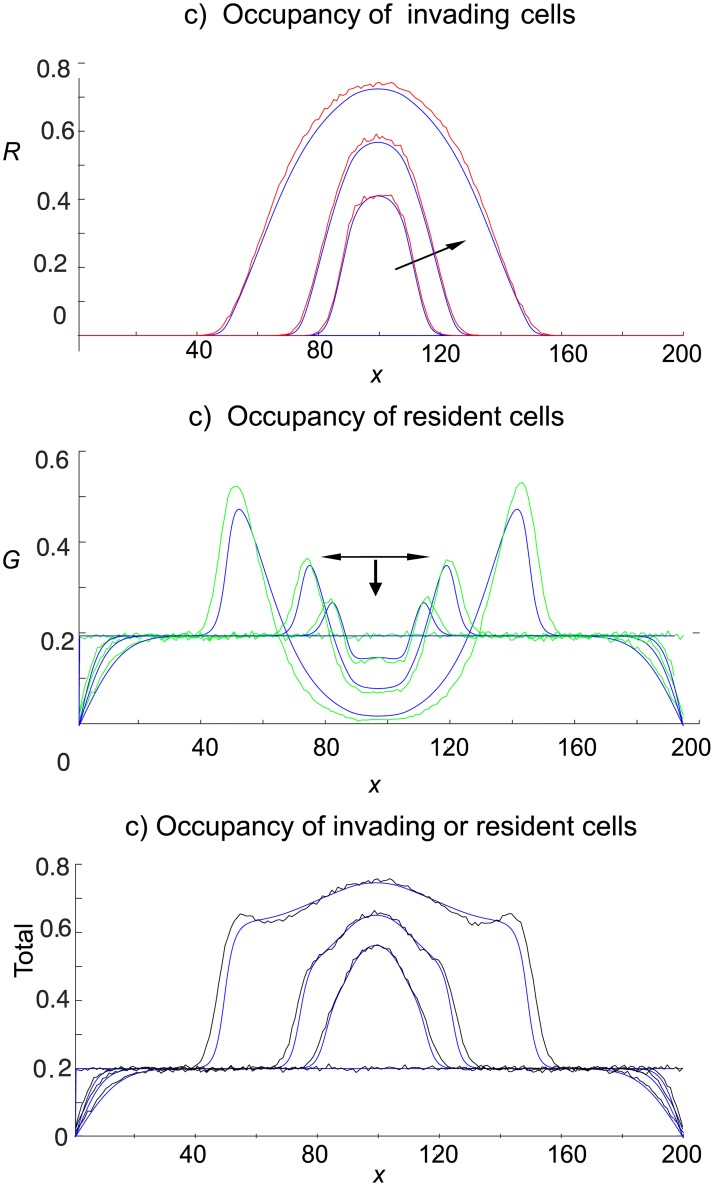
Averaged simulations of the agent–based model compared with the solutions from their mean-field continuum limit PDEs. Red cells are simple shovers and green cells are gentlemen. Red cells enter the domain in the 2D band in the centre of the lattice, and invasion occurs at a rate of four attempts per time-step (which corresponds to an entry probability P_entry_ = 0.01). The initial concentration of green cells is *c*_*G*_ = 0.2. Blue curves are PDE solutions. Simulation results are given at *t* = 0, 50, 100, 250. Simulations are averaged over 300 realisations of the agent–based model. The red cells shoving the green cells leads to crowding of the green cells to the left and right of the invasion region. Site occupancy exceeds 0.65 for much of the lattice by time *t* = 250. Arrows show increasing time.

In Fig 10 we reduce the initial density of green cells from *c*_*G*_ = 0.6 to *c*_*G*_ = 0.2 and obtain a reasonably good agreement between the averaged agent–based simulations and the mean-field derived PDE system (derived in Eqs (7) and (8)), though we note that the continuum equations underestimate the averaged green cell occupancy at the edges of the invasion region where the simulations display an averaged occupancy above 0.5.

It may be noted that Penington et al. [23] have given a general discussion of the derivation via mean-field arguments of parabolic partial differential equations that describe approximately the continuum limit of a broad class of agent-based models. There have been many numerical tests of the agreement between averages of large numbers of *in silico* realisations of specific agent-based models (with or without proliferation) and the predictions of the associated mean-field model (see, e.g. [12, 13, 19, 20]). Although the agreement is often good, the mean-field predictions can become inaccurate if the local interactions between agents have a strong mutual adhesion component that promotes clumping [24], or if proliferation events occur too frequently compared to motility events. Alternatives to the mean-field approach that can improve the concordance of the approximate continuum limit predictions with simulation data from agent–based models have been discussed by Baker and Simpson [25] and by Davies et al. [26].
